# A deregression-based approach to integrate multitrait information into a single pooled phenotype for genetic evaluation

**DOI:** 10.1186/s12711-026-01096-z

**Published:** 2026-10-22

**Authors:** Alice Markey, Nicolas Gengler

**Affiliations:** TERRA Teaching and Research Center, Gembloux Agro-Bio Tech, University of Liège, Gembloux, 5030 Belgium

## Abstract

**Background:**

Genetic evaluation of complex traits is often challenged by heterogeneous, incomplete or costly phenotyping. Many biological processes require multiple traits to be characterized. Moreover, combining several traits becomes difficult when the configuration of available traits is complex (e.g., increasing number, complex missing patterns), leading to more demanding computations. To overcome these limitations, a deregression-based pooling method was developed to pre-process multitrait information into a single pseudo-phenotype of the target trait (i.e., a pooled phenotype). This method was assessed in a terminal crossbreeding situation in pigs.

**Results:**

The behavior of estimated breeding values and the accuracy of reliabilities systems were studied on different datasets (i.e., different data availability scenarios - a real and 3 simulated). Three modeling steps were used: (1) a multitrait model generating input for deregressions, (2) an adjusted scalar deregression, combining information into a pooled phenotype using (3) a single trait model applied to this pooled phenotype to assess how these results match the multitrait predictions from step (1). In all data structure scenarios, unadjusted deregression underestimates breeding values and distorts reliabilities. In contrast, applying expansion and reliability-alignment factors as adjustments restored the distributions and scale of estimated breeding values, yielding a near-perfect agreement with the multitrait model. Across all simulated scenarios, including the one with the complete absence of the target trait, the adjusted deregression process achieved the highest correlations between true and estimated breeding values.

**Conclusions:**

The deregression-based process provides an effective and flexible approach for combining heterogeneous multitrait information into a unique pooled phenotype in a single trait setting, while maintaining genetic scale and reliability of the target trait in a multiple trait setting. Unlike classical deregression, the adjusted deregression process prevents overshrinking, maintains accuracy and reproduces the behavior of a multitrait evaluation. This pre-processing would be a practical solution for integrating complex or partially recorded traits into routine genetic evaluation pipelines. This method is applicable even if the main target trait is limited or missing, provided that moderately to highly correlated traits are available. The strategy could be extended to other situations as long as any double-counting can be avoided.

## Background

Genetic evaluation of complex phenotypes in animal breeding is often limited by the infrastructure, cost, sampling difficulties, limited staff for data collection, or scarcity of direct or complete phenotypes. Frequently, no single trait fully adequately represents the underlying biological process, requiring the combination of partially informative traits that approximate the phenotype of interest. This challenge arises across multiple fields. Physiological or metabolic indicators (e.g., ketosis) capture only part of health [1], while behavioral indicators inform different well-being aspects without providing an exhaustive representation [2, 3]. Phenotypic responses to environmental stressors describe a particular dimension of adaptability or resilience. Environmental performance, such as emissions or system efficiency, can be measured by diverse methods, each targeting distinct dimensions [4]. Product-quality attributes, like meat flavor or texture, require multiple analyses through, for example, volatile organic compounds [5–7]. Finally, sensor-based phenotypes generated for Precision Livestock Farming systems produce multiple correlated signals reflecting potentially the same biological state [8]. Additionally, these traits are often recorded on only a subset of animals, resulting in incomplete and uneven datasets [9]. This can happen in experimental to routine evaluation switches, changes in equipment, innovation or evolution in systems as the use of alternative analysis methods [5, 6]. In the case of complex traits, phenotyping is often based on diverse –omic technologies and can lead to large databases and complex relationships among traits [9]. In theory, multitrait (MT) models, using appropriate strategies [10, 11], are able to integrate large numbers of traits, measurements or proxy variables [12]. These strategies can refer to, for example, reducing dimensionality, stabilizing estimates and make MT models scalable to obtain fast and stable convergence [10–12]. However, their practical application is often constrained in real‑world conditions due to computational and conceptual challenges, particularly the difficulty of estimating large covariance structures [13]. In general, as the number of traits increases, model specifications and computations become more demanding. Current solutions, such as improved dimensionality reduction methods, imputation of missing traits to allow canonical transformation [14] or Bayesian sparse factor analysis [12], only partially address these issues and frequently do not preserve genetic scale or full information. Selection index-based approaches were proposed [7] to combine phenotypes. However, these have problems to handle different missing value patterns. Moreover, all these methods do not generate a reusable combined phenotype. Even though Markey et al. [7] proposed an innovative approach to get a pooled phenotype based on selection index, regardless of the target trait in this pooling process, this approach does not address the issue of partial availability of data for some traits. Therefore, a methodological gap remains in approaches able to integrate heterogeneous and incomplete information from multiple correlated traits, while allowing pooling the information into one trait without compromising computational efficiency.

Together, these constraints hinder efficient integration of heterogeneous information and motivate the development of alternative and more flexible approaches. Boar taint exemplifies these challenges. While conceptually simple (consumer perception), its chemical basis is complex, involving multiple compounds [15, 16]. The target trait (i.e., in this case, sensory scores) is routinely assessed by trained evaluators, while chemical measurements are costly and explain only ~ 50% of human perception [7]. Numerous proxy traits exist of which skatole and androstenone are the best known. Often these traits are more heritable than sensory scores, but each captures only a portion of the phenotypic and genetic variabilities of the boar taint phenotype [7, 15, 17]. Furthermore, due to some of the issues explained above, skatole and androstenone quantifications are not always available [7, 18, 19].

Based on this and on similar situations found in other contexts, we suggest that genetic evaluations could be improved by a priori pooling partially informative traits to create a pooled phenotype for further use. Such an approach would provide a scalable and practical framework to integrate heterogeneous information. Its setup should be flexible by generating pseudo-records, that can be accumulated over time independently, generated in different circumstances (e.g., allow different sets of traits) and that can be directly combined with observed records. This system should facilitate the inclusion of a new pooled phenotype with several different dimensions and traits, while remaining robust and eliminating the need for full MT evaluation. The pooled phenotype represents the target trait in the evaluation, enhanced by other traits. For an effective pooled phenotype, the genetic information must be combined across correlated traits, the correct scale of genetic variance of the target trait must be preserved and consistency must be maintained between expected and observed reliabilities (REL) of the target trait. The pooled phenotype must also be compatible with genetic evaluation pipelines. Ultimately, the pooled phenotype can be defined as a pseudo-phenotype of the target trait enhanced by other correlated traits while preserving the genetic variance of the target trait.

Therefore, the objectives of this study were (1) to develop a novel method for pooling MT information into a single pseudo-phenotype of the target trait (i.e., the pooled phenotype) suitable for genetic evaluation, and (2) to evaluate the accuracy and feasibility of this approach using both real and simulated datasets in the context of boar taint in terminal crossbreed pigs.

## Methods

### Models

In this study, different variants of the same mixed model were used. First of all, an MT model was applied to three traits. Then, the model was adapted to a single trait evaluation. In the subsection of “Datasets”, a simplified version of the model was used to generate simulated data.

The linear mixed animal model used in different variants in this study can be represented as:

1 $$\:\mathbf{y}=\mathbf{X}\mathbf{b}+\mathbf{Z}\mathbf{a}+\mathbf{e},$$

where $\:\mathbf{y}$ is the vector of phenotypic observations as described below, $\:\mathbf{b}$ is the vector of fixed effects represented by contemporary groups and a regression on metabolic weight, $\:\mathbf{a}$ is the vector of random genetic effects, $\:\mathbf{e}$ is the vector of random residuals, and $\:\mathbf{X}$ and $\:\mathbf{Z}$ are the respective incidence matrices.

For the MT model, $\:\mathbf{y}$ represented the traits sensory score, skatole and androstenone (Traits 1, 2, 3, respectively) grouped within records. All other effects and incidence matrices were organized according to the structure of $\:\mathbf{y}$. The additive genetic and residual effects were considered to follow multivariate Gaussian distributions $\:{\mathbf{a}}\sim N({\bf 0},{\mathbf{A}}\otimes\:{{\mathbf{G}}}_{0})\:$and $\:{\mathbf{e}}\sim N({\bf 0},{\mathbf{I}}\otimes\:{{\mathbf{R}}}_{0})\:$, where $\:{\mathbf{G}}_{0}$ and $\:{\mathbf{R}}_{0}$ are the additive genetic and residual (co)variances matrices among traits, respectively, $\:\mathbf{A}$ is the relationship matrix based on pedigree, $\:\mathbf{I}$ is an identity matrix.

In order to evaluate the pooled phenotype, the linear mixed model was adapted to a weighted ST animal model for the two following points: (1) The vector of phenotypic observations $\:\mathbf{y}$ contained the pooled phenotype; (2) The additive genetic and residual effects were considered to follow the distributions $\:{\mathbf{a}} \sim N({\bf 0},{\mathbf{A}}{\sigma\:}_{a}^{2})$ and ${\mathbf{e}} \sim N({\bf 0},{f{\mathbf{W}}}^{-1}{\sigma\:}_{e}^{2})$ where $\:{\sigma\:}_{a}^{2}$ and $\:{\sigma\:}_{e}^{2}$ are the additive genetic and residual variances, respectively, $\:\mathbf{A}$ is the relationship matrix based on pedigree, $\:\mathbf{W}$ is a diagonal matrix containing the weights. All definitions of fixed effects stayed the same.

Based on the MT model, the variance components were estimated using the BLUPF90+ program with EM-REML [20]. Convergence was considered achieved at $\:1{0}^{-12}$. The estimated variances of the target trait were used in the ST model. The EBV and REL were also calculated using the BLUPF90+ program. For a given animal* i*, its $\:RE{L}_{i}$ was derived from the obtained prediction error variance ($\:PE{V}_{i}$) of its $\:EB{V}_{i}$ according to Aguilar et al. [21] as

2 $$\:RE{L}_{i}=1-\left[PE{V}_{i}/\left({\sigma\:}_{a}^{2}\right(1+{F}_{i}\left)\right)\right],$$

where $\:{\sigma\:}_{a}^{2}$ is the genetic variance and $\:{F}_{i}$ is the inbreeding coefficient of animal$\:\:i$.

### Deregression process

#### Computational approach

Using an MT model and corresponding variance components (obtained from literature or estimated), estimated breeding values (EBV_MT) and reliabilities (REL_MT) can be calculated for all traits, including the target trait. The EBV_MT contain information from the own records of a given animal, its relatives and all genetically correlated traits included in the MT evaluation. When original phenotypic data for genetic evaluation is not available, EBV deregression is widely used for deriving pseudo-phenotypes. These pseudo-phenotypes can then be evaluated to regenerate EBV using an ST model. If the deregression correctly exploits all the information contained in the EBV_MT, the EBV regenerated from these pseudo-phenotypes should remain equivalent to the original EBV_MT [22–24]. In our study, the deregression of EBV_MT was specifically applied to the target trait in order to combine information from all correlated traits. This targeted use of deregression, although conceptually coherent, remains uncommon.

This pseudo-phenotype, called “deregressed proof” (DRP), represents the non-parental part of the EBV of an animal. The DRP reflects the Mendelian sampling term, i.e., the EBV without parental contributions (removed) and the potential part of information from progeny when available (which is not the case in a terminal cross context). During the deregression step, the Mendelian sampling term is expanded to counteract the shrinkage applied during breeding value estimation. Each DRP is accompanied by an Effective record contribution (ERC), or in the case of a sire model, an Effective daughter contribution (EDC), which quantifies how much information supports that DRP. In this study, we used an animal model type of implementation and the Mendelian‑sampling‑based component of the ERC (i.e., dERC) as the weight associated with each DRP [25]. dERC were used rather than the full ERC because dERC reflects the individual information weighting each DRP discounted for parental contributions.

The process of deregression should create reversibility [26]. In our case, we will refer to this as extended reversibility. The concept of reversibility can be defined as the ability of a deregression process to generate DRP that lead to the original EBV. When used as pseudo‑records in a subsequent evaluation (i.e., ST model), with dERC as their weights, the resulting EBV (EBV_DRP) should closely reproduce the original EBV_MT for the target trait. Indeed, the EBV_MT incorporate information from correlated traits, therefore, the DRP derived from EBV_MT also embed this MT information. Moreover, the re-estimated reliability (REL_DRP) should remain aligned with REL_MT.

#### Basic theory

Many different deregression methods exist. From the simplest ones proposed by Garrick et al. [27] (e.g., dividing each EBV by its REL) such as those used in early genome-wide association studies (GWAS) and genomic prediction studies to more complicated approaches such as the matrix deregression method [24] used by INTERBULL in the context of international genetic evaluations. More detailed descriptions and comparisons of these methods were provided by Calus et al. [23]. In the case of terminal crosses, as here, the scalar approach presented by Bonifazi et al. [22] is particularly well suited. We will therefore base our developments on these equations, but adaptations to non-scalar deregression methods, such as matrix deregression methods, are rather straightforward.

In the context of this study, the total ERC for a given animal * i* with records can be approximated using

3 $$\:ER{C}_{i}=\lambda\:\frac{RE{L}_{i}}{1-RE{L}_{i}}\:\:\:\mathrm{w}\mathrm{i}\mathrm{t}\mathrm{h}\:\lambda\:=\frac{{\sigma\:}_{e}^{2}}{{\sigma\:}_{a}^{2}},$$

where $\:RE{L}_{i}$ is the total reliability associated with the EBV of animal * i*. The total ERC for animal$\:\:i$ is the sum of contributions from parents (* PA*), own (* o*) and progeny (* p*). Contributions from parents of animal * i* can be computed using

4 $$\:RE{L}_{PA,i}=\frac{RE{L}_{sire}+RE{L}_{dam}}{4},$$

5 $$\:ER{C}_{PA,i}=\lambda\:\frac{RE{L}_{PA,i}}{1-RE{L}_{PA,i}},$$

where the contribution linked to * o* and * p* is given by

6 $$\:dER{C}_{i}=ER{C}_{i}-ER{C}_{PA,i}.$$

Based on * dERC*, corresponding reliabilities can be computed for each animal * i* as follows:

7 $$\:RE{L}_{i\left(o+p\right)}=\frac{dER{C}_{i}}{dER{C}_{i}+\lambda\:}.$$

The value of REL is equivalent to the theoretical regression coefficient in the context of mass selection transforming a phenotypic deviation into a genetic one [28]. Therefore, in the context of Mendelian sampling, the DRP value of animal * i* can be obtained by deregressing the EBV of $\:i\:$as follows:

8 $$\:DR{P}_{i}=P{A}_{i}+\frac{EB{V}_{i}-P{A}_{i}}{RE{L}_{i\left(o+p\right)}},$$

9 $$\:P{A}_{i}=\frac{EB{V}_{sire}+EB{V}_{dam}}{2}.$$

Up to this point the derivation of the DRP element for individual$\:\:i$ follows the outline presented in Bonifazi et al. [22]. However, there are two hidden issues. The first issue concerns the DRP as predicted values, which inherently involve two sources of error. On the one hand, every prediction comes with an associated prediction error, yet the variance of the predicted values does not reflect the uncertainty inherent to each prediction. Consequently, the variance of the predictions will always be smaller than (or equal to) that of the underlying variable they are intended to represent. On the other hand, while the deregression process can be adapted to the model in which the prediction will be used (in our case, a ST model), it does not reflect the model generating the EBV (in our case, an MT model). This mismatch is also expected to create a difference in genetic variance, since the contributions of correlated traits must be considered. The second issue concerns the dERC which must accurately represent the information content of the DRP. Indeed, the correction of DRP explained above may create incoherent REL because dERC and DRP are interdependent. In fact, the REL are derived from the inverse of the left-hand-side. Therefore, REL are related to the dERC. However, EBV are derived from both the left-hand-side (i.e., dERC related) and the right-hand-side (i.e., dERC and DRP related). This creates a complex and non‑trivial relationship between predicted EBV and their REL with dERC and DRP leading to the need to adjust both jointly in an iterative process to restore coherence between EBV, REL, DRP and dERC.

#### Adaptations

The first issue can be translated to the rationale that DRP are too strongly regressed. The deregression step could be adjusted following VanRaden et al. [29], supported by Cole and VanRaden [30], who showed the conceptual similarity between deregression and expansion of a predicted record. The theoretical solution to this problem lies in the expansion of predicted DRP of the target trait by adjusting the deregression step by using a factor $\:\omega\:$ to generate:

10 $$\:DR{P}_{i}^{*}=P{A}_{i}+\frac{EB{V}_{i}-P{A}_{i}}{\omega\:RE{L}_{i\left(o+p\right)}},$$

where $\:1/\omega\:$ is representing the expansion factor analogue to the one proposed by VanRaden et al. [29]. They outlined that the empirical expansion factors can be computed as ratios of standard deviations (SD) of the expected genetic effects divided by the SD of observed genetic effects, i.e., the SD of EBV from MT divided by the SD of EBV from ST (based on DRP of the animals with records). While VanRaden et al. [29] based their expansion factor on sire effects, we based it on the population of animals with records, given that an animal model was used here. A second adaptation compared to the method used by VanRaden et al. [29] lies in the need to use iterations. As a matter of fact, adjusted DRP still generates EBV showing suboptimal expansion because SD of EBV_DRP remain different from SD of EBV_MT of the target trait. However, by recomputing $\:\omega\:$ appropriately in a recursive manner, the expected expansion converged towards equal SD of EBV_DRP and EBV_MT. For the second issue, an empirical correction for an animal * i* is to adapt the weight $\:{dERC}_{i}\:$in order to generate mixed model equations (i.e., coefficient matrix) that yield the correct REL. The least square part of these equations is linked to $\:{dERC}_{i}$ (i.e., approximately proportional). The hypothesis is that the values for REL_MT and REL_DRP, after adjustment, for an animal * i*, should be equivalent. In order to achieve this, $\:RE{L}_{i}$ (from MT) for each animal $\:i\:$was multiplicatively adjusted using the reliability-alignment factor $\:\gamma\:$:

11 $$\:RE{L}_{i}^{*}=\gamma\:RE{L}_{i},$$

where

12 $$\:\gamma\:\:=\:(SD\:of\:REL\_MT)/(SD\:of\:REL\_ST).$$

Finally, using $\:RE{L}_{i}^{*}$, $\:dER{C}_{i}^{*}$ was obtained as follows:

13 $$\:dER{C}_{i}^{*}=ER{C}_{i}^{*}-ER{C}_{PA,i},$$

where

14 $$\:ER{C}_{i}^{*}=\lambda\:\frac{RE{L}_{i}^{*}}{1-RE{L}_{i}^{*}}.$$

The expected reliability-alignment factor converged towards equal SD of REL_DRP and REL_MT.

Both adjustment factors were computed jointly and applied consecutively within the same iteration of the deregression process. The deregression step was followed by a re-evaluation of the pooled phenotype using the ST model described above (i.e., with BLUPF90+ program [20]). The EBV and REL obtained using $\:{DRP}^{*}$ and $\:{dERC}^{*}\:$will hereafter be referred to as EBV_ADJ_DRP and REL_ADJ_DRP, respectively. The equalities between SD of EBV_MT and EBV_DRP_ADJ, and SD of REL_MT and REL_DRP_ADJ, was iteratively verified. From a conceptual point of view, adjusting the DRP serves to restore the correct genetic variance, while adjusting the dERC ensures that the average information content is properly aligned. The factors $\:1/\omega\:$ and $\:\gamma\:$ were readjusted and reapplied in the deregression step. In addition to the SD equalities verification, convergence of the factors $\:1/\omega\:$ and $\:\gamma\:$ was assessed using the relative squared change in correlations between successive iteration for the EBV and the REL. This indicator was defined as $\:{\left(\left({r}_{t}-\:{r}_{t-1}\right)/{r}_{t-1}\right)}^{2}$, where $\:{r}_{t}$ is the correlation at iteration * t*, between either the EBV or the REL from the MT and the ST models. Convergence was assumed when both values (i.e., correlations for EBV and REL respectively) became lower than $\:1{0}^{-7}$. As a result, the repeated adapted deregression successfully yielded identical SD for EBV_MT and the EBV_ADJ_DRP, and for SD for REL_MT and REL_ADJ_DRP. The deregression step was implemented using SAS 9.4 software [31].

### Datasets

Two datasets were used to develop and to test the proposed pooling method. The first dataset, called “real dataset”, consisted of observed measurements of sensory score, skatole and androstenone, collected between 2020 and 2025 through the NoWallOdor project on boar taint. The second dataset, called “simulated dataset”, included the same three traits, extended to all terminal crossbred animals included in the Walloon pig genetic evaluations testing program in Belgium (since 2007). To obtain a complete dataset covering the entire period, the simulations assumed that all slaughtered animals initially had records.

#### The real dataset

Boar taint phenotypes were available for 2169 Piétrain × Landrace crossbreeds (animals without the target trait (i.e., missing sensory scores and incomplete pedigree) were excluded). They were raised for approximately 28 weeks (average weight of 120 ± 15 kg) in batches of around 100 animals born on the same farm and then fattened in two farms. Each batch × farm combination constitutes a contemporary group. Boar taint measurements (sensory analysis (*n* = 2169); quantification of skatole and androstenone (*n* = 388)) were performed on neck fat collected at the slaughterhouse and stored at − 20 °C. A trained assessor evaluated boar taint according to a three-point scale. The compounds were quantified after methanol extraction and UHPLC-MS/MS analysis, using an ISO17025-accredited laboratory. More details can be found in Markey et al. [7]. These data were transformed and standardized. The sensory score (0-1-2) was converted into a Snell score to approximate a continuous distribution [7, 17, 32, 33]. The quantifications were transformed using a base-10 logarithm to approximate normality in formally skewed distributions [7, 17, 34]. Finally, the three traits were standardized by subtracting the mean and dividing by the standard deviation calculated for the analyzed population and will be hereafter called Trait 1 (sensory score), Trait 2 (skatole) and Trait 3 (androstenone). The three-generation pedigree was extracted (*n* = 2672). These data were used to estimate the variance components subsequently used in the simulation.

#### Extended simulated dataset

In research, and sometimes in routine as well, it is common to encounter data that is missing or scattered, or to use a new target trait that has become available (i.e., without records). The real dataset being rather small and the pattern of observed and missing traits being fixed by its configuration, an extended simulated dataset was generated from the whole pedigree of the Walloon pig genetic evaluation system. This pedigree contained 9590 crossbred animals for which boar taint (i.e., data for sensory score, skatole and androstenone) could be considered as retrospectively available.

The simulation approach explained below follows the strategy outlined by Van Vleck [35]. This strategy was adapted to allow for a categorial trait and was adjusted so that the variance components observed on the simulated dataset matched those in the real dataset. To achieve this, steps 2 to 5. described below were performed iteratively. The computations were programmed in GNU Octave [36] and BLUPF90+ [20] for the variance component estimations. The following simulation procedure was used:

- The complete pedigree available through the testing program of boars in Wallonia was used independently of the boar taint study, i.e. included in the real data set. All crossbred offspring tested since 2007 in the program were identified from the complete pedigree. A cleaning (e.g., duplicate animals and crossbred animals with incomplete pedigrees exclusion) of all tested animals was performed obtaining 9590 animals. Then, their ancestors were extracted to create a three-generation pedigree (*n* = 11,615 animals). The simulation was based on this pedigree to obtain a simulated dataset structure as close as possible to the real data structure. Animals were sorted from the oldest ancestors to the most recent crossbred descendants. Finally, the additive numerator relationship matrix $\:\mathbf{A}$ between these 11,615 animals was built using the tabular method first described by Emik and Terrill [37].
- Simulated random effects were sampled from multivariate normal distributions using $\:\mathbf{a} \sim N(\mathbf{0},\mathbf{A}\otimes\:{\mathbf{G}}_{0})$ and $\:\mathbf{e} \sim N(\mathbf{0},\mathbf{I}\otimes\:{\mathbf{R}}_{0})\:$, where $\:{\mathbf{G}}_{0}$ and $\:{\mathbf{R}}_{0}$ are the 3 × 3 additive genetic and residual (co)variances matrices among traits, respectively, $\:\mathbf{A}$ is the relationship matrix based on pedigree, and $\:\mathbf{I}$ is an identity matrix. At the first iteration, used variance components for the three traits were those estimated in the real data.
- In the initial round (round 0): Cholesky decompositions of the pedigree relationship matrix $\:\mathbf{A}={\mathbf{L}}_{\mathbf{A}}{{\mathbf{L}}_{\mathbf{A}}}^{{\prime\:}}$, of the genetic $\:{\mathbf{G}}_{0}={\mathbf{L}}_{{\mathbf{G}}_{0}}{{\mathbf{L}}_{{\mathbf{G}}_{0}}}^{{\prime\:}}$ and of the residual $\:{\mathbf{R}}_{0}={\mathbf{L}}_{{\mathbf{R}}_{0}}{{\mathbf{L}}_{{\mathbf{R}}_{0}}}^{{\prime\:}}$ matrices were used to facilitate simulation of the actual pedigree and trait structure. Vectors $\:\mathbf{a}$ and $\:\mathbf{e}$ were generated using $\:\mathbf{a}={\mathbf{L}}_{\mathbf{A}}\otimes\:{\mathbf{L}}_{{\mathbf{G}}_{0}}{\mathbf{s}}_{\mathbf{a}}$ and $\:\mathbf{e}=\mathbf{I}\otimes\:{\mathbf{L}}_{{\mathbf{R}}_{0}}{\mathbf{s}}_{\mathbf{e}}$, where $\:\mathbf{a}$ is the vector of random simulated genetic effects, $\:\mathbf{e}$ is the vector of random simulated residuals, $\:{\mathbf{s}}_{\mathbf{a}}$ and $\:{\mathbf{s}}_{\mathbf{e}}$ are vectors of simulated random standard normal variables using the random function randn().
- Based on Cholesky decomposition of the re-estimated genetic and residual $\:{\mathbf{R}}_{t}={\mathbf{L}}_{{\mathbf{R}}_{t}}{{\mathbf{L}}_{{\mathbf{R}}_{t}}}^{{\prime\:}}$ matrices, at any given round * t*, the following transformation matrices of the data for the next round $\:{\mathbf{T}}_{{\mathbf{G}}_{t}}={\mathbf{L}}_{{\mathbf{G}}_{0}}{\left({\mathbf{L}}_{{\mathbf{G}}_{t}}\right)}^{-1}$ and $\:{\mathbf{T}}_{{\mathbf{R}}_{t}}={\mathbf{L}}_{{\mathbf{R}}_{0}}{\left({\mathbf{L}}_{{\mathbf{R}}_{t}}\right)}^{-1}$ were defined. In each following round (round * t*): Adjusted vectors $\:\mathbf{a}$ and $\:\mathbf{e}$ were computed given the results from step 4. using $\:\mathbf{a}={\mathbf{L}}_{\mathbf{A}}\otimes\:\left({\prod\:}_{i=t}^{1}{\mathbf{T}}_{{\mathbf{G}}_{i}}\right){\mathbf{L}}_{{\mathbf{G}}_{0}}{\mathbf{s}}_{\mathbf{a}}$ and $\:\mathbf{e}=\mathbf{I}\otimes\:\left({\prod\:}_{i=t}^{1}{\mathbf{T}}_{{\mathbf{R}}_{i}}\right){\mathbf{L}}_{{\mathbf{R}}_{0}}{\mathbf{s}}_{\mathbf{e}}$, where $\:{\prod\:}_{i=t}^{1}{\mathbf{T}}_{{\mathbf{G}}_{i}}$ and $\:{\prod\:}_{i=t}^{1}{\mathbf{T}}_{{\mathbf{R}}_{i}}$ were computed based on results from step 5. in round 1 to current round * t*. The simulated vectors $\:{\mathbf{s}}_{\mathbf{a}}$ and $\:{\mathbf{s}}_{\mathbf{e}}$ remaining identical by using the same seed. Consequently, changes observed across rounds are solely attributable to modifications of the covariance structures.

Then, the simplified generic MT model was used to compute $\:\mathbf{y}=\mathbf{Z}\mathbf{a}+\mathbf{e},$ where $\:\mathbf{y}$ is the vector of simulated records (Traits 1, 2, 3 representing respectively sensory score, skatole and androstenone) and $\:\mathbf{Z}$ is the respective incidence matrix. Fixed effects, consisting of a single contemporary group and a regression on a constant metabolic weight, were implicitly simulated by setting their effect to zero allowing to maintain a coefficient matrix structure as close as possible to the real dataset. As this was equivalent to not simulating these effects, we could use the simplified model above.

3. In order to reproduce the discrete structure of Trait 1 (i.e., the Snell sensory score trait) observed in the real dataset, the simulated continuous values were converted into Snell scores. The boundaries of each Snell score were defined so that the proportion of simulated observations followed the empirical frequency distribution of each original Snell sensory score from the real dataset. All three simulated traits were then phenotypically standardized as was done in the real data.
4. An MT model with fixed effects defined as a single contemporary group and the regression on an average metabolic weight was used to re-estimate variance components of the linear mixed animal model presented in the Models section.
5. The procedure was iteratively run until re-estimated variance components became stable. Variance components estimated, at any given round $\:t\:$($\:{\mathbf{G}}_{t}$ and $\:{\mathbf{R}}_{t}$), on the simulated dataset were compared to initial estimates $\:{\mathbf{G}}_{0}$ and $\:{\mathbf{R}}_{0}$ using Frobenius norms of the differences between genetic and residual (co)variance and correlation matrices. More details are available in the following “Comparison of results” subsection.

To study the effect of heterogeneous phenotype configurations, three datasets were derived from this simulated dataset by varying the availability of the three traits. Depending on the case, some combinations of traits were fully measured while others contained only a subset of measurements. In such cases, the target trait could be absent. To avoid bias, the data were randomly eliminated, using the following scenarios:

##### • Case 1:

Data for Traits 1, 2 and 3 were available for all crossbred animals (*n* = 9590) (i.e., original simulated dataset);

##### • Case 2:

Data were available for 90% of crossbred animals for Trait 1 and 15% for Traits 2 and 3, and each animal had at least one observation for Trait 1 or Traits 2 and 3;

##### • Case 3:

Data were available for all crossbred animals for Traits 2 and 3, and no data were available for Trait 1.

### Comparison of results

Comparisons of initial and re-estimated covariance and correlation matrices were done using Frobenius norms on the relative difference (i.e., error for re-estimated matrix at round * t*) between matrices as proposed by Misztal et al. [38], where $\:err\left({\mathbf{M}}_{t}\right)={||{\mathbf{M}}_{t}-{\mathbf{M}}_{0}||}_{F}/{||{\mathbf{M}}_{0}||}_{F}$ with $\:{||\mathbf{M}||}_{F}=\sqrt{{\sum\:}_{i=1}^{3}{\sum\:}_{j=1}^{3}{m}_{ij}^{2}}$ being the Frobenius norm of a given matrix $\:\mathbf{M}$, where $\:i\:$is the row, * j* is the column number and * m* is matrix element at that position. This procedure allowed to evaluate the divergences between covariance components as a way to evaluate the success of the process to generate extended synthetic data. As Frobenius norms work best for matrices with similar diagonal elements, we used both (co)variance and correlation matrices, the diagonal elements of the latter being equal.

The comparison of the EBV from the three systems (EBV_MT, EBV_DRP and EBV_ADJ_DRP) was based on descriptive statistics, regressions and plots illustrating the distribution of results of the (adjusted) deregression process to the reference model, MT. Results are reported for crossbred animals (i.e., with phenotype) and sires of crossbred animals called G1 for real and simulated datasets which is the group (i.e., the group of sires) being the most relevant animals in the context of genetic evaluation for boar taint.

Extending from basic theoretical considerations [21, 28], we postulated that REL of EBV can be compared to the squared correlation between true breeding values and EBV in order to assess accuracy and the coherence between reliability and realized predictive performance across MT, DRP and ADJ_DRP for the three cases. We calculated the correlation between true breeding values and predicted EBV (* r*), which represent the realized accuracy of each system and its square ($\:r^{2}$) which represents the expected mean reliability [21, 28]. Comparing $\:r^{2}$ with the reported mean model reliabilities ($\:\overline{{REL}}$) through |$\:\overline{{REL}}\mathrm{-}\text{}r^{2}$| quantifies how closely the expected information content of the evaluations matches its actual predictive ability. Comparing these three metrics across MT, DRP and ADJ_DRP for the three cases provided a concise and consistent theoretical basis for evaluating whether DRP or ADJ_DRP reproduce the accuracy and reliability structure of the MT model across datasets. The SAS 9.4 software [31] was used for statistics and graphics.

## Results

### Descriptive statistics of real data

**Fig. 1:**
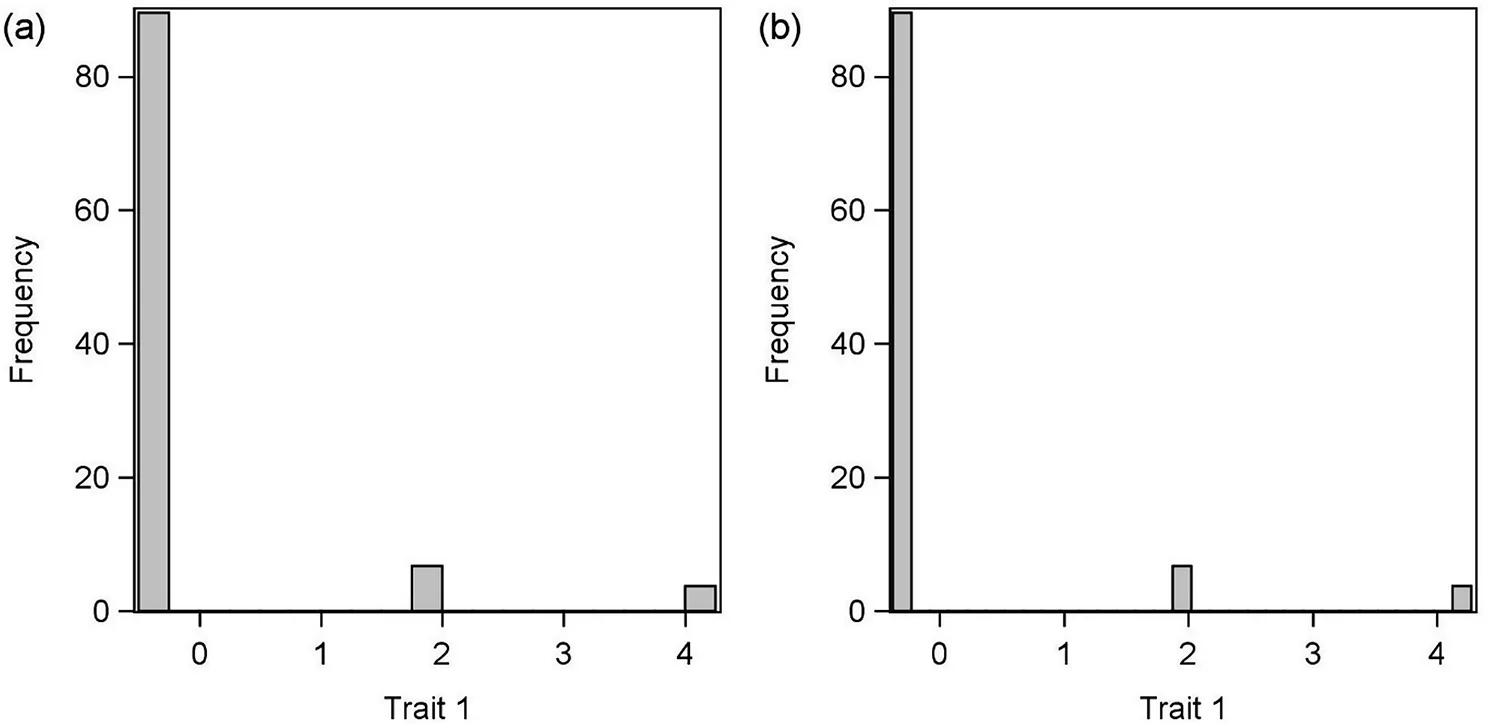
Distribution of Trait 1 in the both datasets. Distribution of Trait 1, (**a**) as observed in the real dataset and (**b**) simulated in the extended dataset

Table 1 summarizes the descriptive statistics of the three original traits prior to any transformation. These values reflect the known imbalance of boar taint phenotyping, where target trait records (i.e., sensory score) are widely available, while chemical measurements are limited to a smaller subset of animals. Table 1 also reports the standardized transformed traits, called hereafter Trait 1 (i.e., target trait), Trait 2 and Trait 3 (Snell for sensory score, log_10_ for skatole and androstenone). As Trait 1 represented sensory score, Fig. 1 gives the distribution of Snell sensory scores into 3 classes.

**Table 1:** Descriptive statistics of the real dataset (original and standardized transformed traits)

Traits	*n*	Mean	Standard Deviation	Minimum	Maximum
Original sensory score	2169	0.14	0.44	0.00	2.00
Original skatole (ppb)	388	263.18	410.56	0.87	3665.29
Original androstenone (ppb)	388	1843.23	1675.11	5.03	9358.00
Standardized sensory score	2169	0.00	1.00	-0.32	4.17
Standardized skatole	388	0.00	1.00	-1.98	1.83
Standardized androstenone	388	0.00	1.00	-3.49	1.48

### Variance components for real and simulated data

Table 2 reports the variance component estimations and the calculated genetic parameters for MT models applied to the real (**a,c,d**) and simulated (**b,e,f**) datasets obtained after 16 iterations. The calculated Frobenius norms of the relative differences were lower than 1% ($\:5.98\times\:{10}^{-3}$ and $\:4.09\times\:{10}^{-3}$ for genetic and residual (co)variance matrices and $\:7.55\times\:{10}^{-3}$ and $\:3.46\times\:{10}^{-4}$ for the genetic and residual correlation matrices). The latter reflects the relative difference of the off-diagonals as diagonal elements are equal for correlation matrices.

**Table 2:** Genetic parameters and (co)variances in real and simulated datasets

	Trait 1	Trait 2	Trait 3
**(a)**			
Trait 1	**0.092**	0.745	0.636
Trait 2	0.216	**0.337**	0.446
Trait 3	0.170	0.464	**0.460**
**(b)**			
Trait 1	**0.092**	0.748	0.630
Trait 2	0.216	**0.338**	0.448
Trait 3	0.170	0.463	**0.461**
**(c)**			
Trait 1	0.091	0.120	0.124
Trait 2	0.120	0.283	0.153
Trait 3	0.124	0.153	0.417
**(d)**			
Trait 1	0.902	0.153	0.113
Trait 2	0.153	0.557	0.242
Trait 3	0.113	0.242	0.490
**(e)**			
Trait 1	0.092	0.121	0.124
Trait 2	0.121	0.285	0.155
Trait 3	0.124	0.155	0.418
**(f)**			
Trait 1	0.911	0.154	0.114
Trait 2	0.154	0.557	0.242
Trait 3	0.114	0.242	0.490

With heritability on the diagonal, genetic correlations (above) and residual correlations (below) in (a) and (b), variances on the diagonal and covariances off-diagonal for (c) genetic (co)variances of the real dataset, (d) residual (co)variances of the real dataset, (e) genetic (co)variances of the simulated dataset, (f) residual (co)variances of the simulated dataset

### Adjustment of deregression

**Fig. 2:**
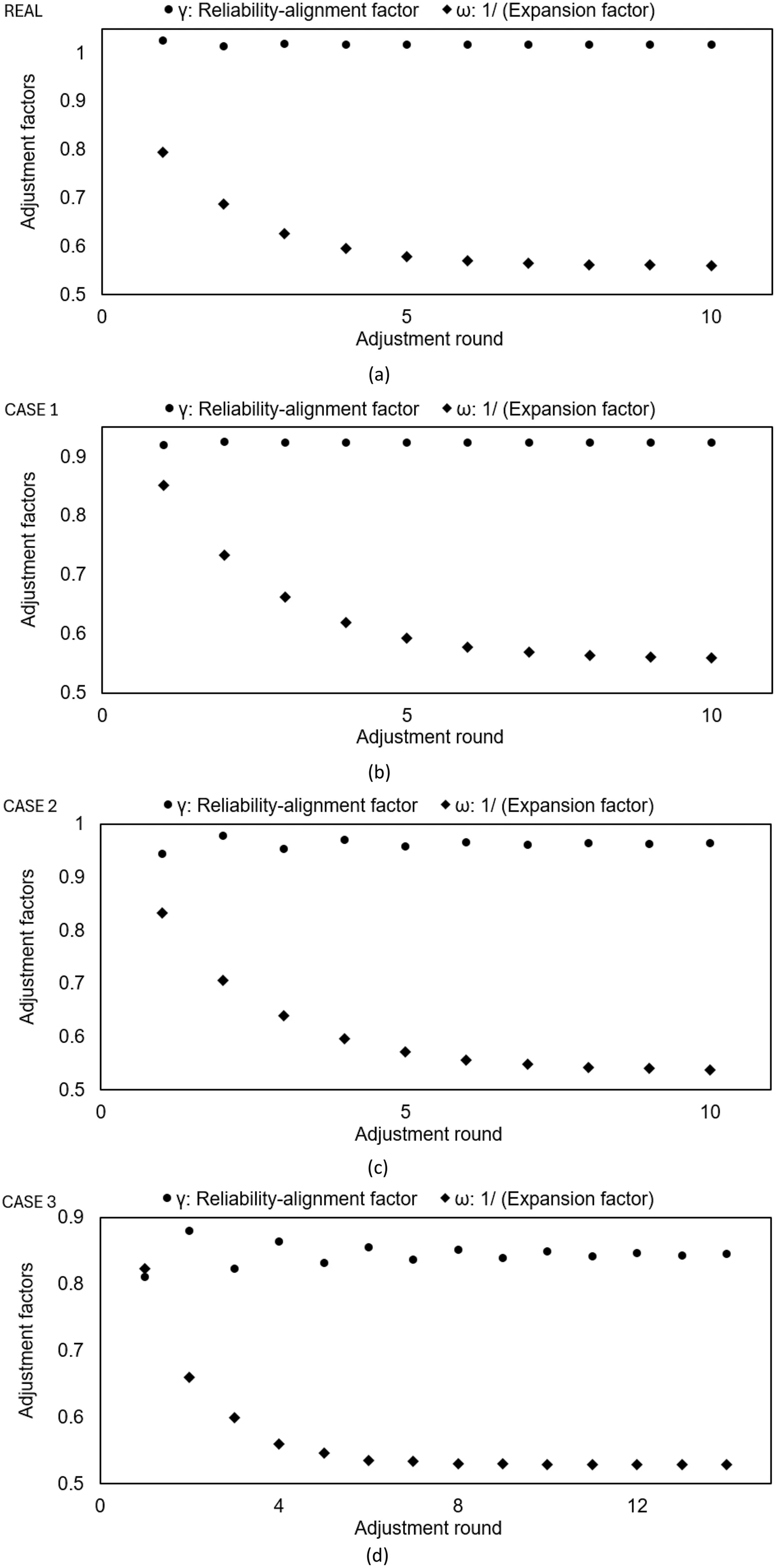
Evolution of the adjustment factors across the iterative adjustment process. Evolution of the adjustment factors ($\:1/\omega\:$,$\:\gamma\:$) for the real dataset (**a**) and cases 1-3 (**b**-**d**) of the simulated dataset

Figure 2 represents the values of the expansion factor ($\:1/\omega\:$) and the reliability-alignment factor ($\:\gamma\:$) for the real dataset and the three cases of the simulated dataset. For the Case 3, as example, the expansion factor ($\:1/\omega\:$) shows an asymptotic decline from 0.82 and decreases rapidly to 0.53. The reliability-alignment factor ($\:\gamma\:$) fluctuates slightly between 0.81 and 0.88 to reach 0.85 at convergence. Factors for other cases and the real dataset shows similar trends. Convergence of adjustment iterations was reached rapidly. The relative squared change in correlations for EBV and REL being below $\:1\:\times\:{10}^{-10}\:$for all datasets after 10 rounds, except for Case 3, where 14 rounds were required for REL to reach $\:7.2\:\times\:{10}^{-7}$, a value considered low enough. In practice, the EBV of DRP were associated to a mean REL value that differed from those obtained in MT or ADJ_DRP, such as for example in the real dataset. For the phenotyped animals, the mean REL_MT value was 0.291, differing to 0.282 with unadjusted deregression and then returned to a value aligned to MT with 0.292 when the adjusted deregression process was applied. For sires, these values were, respectively, 0.411 (REL_MT), 0.405 (REL_DRP) and 0.416 (REL_ADJ_DRP). Other REL means are represented in Table 3.

**Table 3:** Combined accuracy metrics** (***r*, ${r}^{2}$**) **and coherence with reliability of the target trait **(|**$\:\overline{{R}{E}{L}}-\:{r}^{2}$|) for phenotyped animals and sires across simulated cases

	Phenotyped animals					Sires			
	$\:\boldsymbol{r}$	$\:\boldsymbol{r}^{2}$	$\:\overline{\boldsymbol{R}\boldsymbol{E}\boldsymbol{L}}$	|$\:\overline{\boldsymbol{R}\boldsymbol{E}\boldsymbol{L}}-\:\boldsymbol{r}^{2}$|		$\:\boldsymbol{r}$	$\:\boldsymbol{r}^{2}$	$\:\overline{\boldsymbol{R}\boldsymbol{E}\boldsymbol{L}}$	|$\:\overline{\boldsymbol{R}\boldsymbol{E}\boldsymbol{L}}-\:\boldsymbol{r}^{2}$|
Case 1									
MT	0.651	0.424	0.443	0.019		0.734	0.539	0.561	0.022
DRP	0.642	0.412	0.478	0.066		0.727	0.529	0.633	0.104
ADJ_DRP	0.651	0.424	0.432	0.008		0.734	0.539	0.585	0.046
Case 2									
MT	0.563	0.317	0.339	0.022		0.639	0.408	0.460	0.052
DRP	0.557	0.310	0.372	0.062		0.634	0.402	0.504	0.102
ADJ_DRP	0.562	0.316	0.347	0.031		0.638	0.407	0.473	0.066
Case 3									
MT	0.609	0.371	0.370	0.001		0.683	0.466	0.469	0.004
DRP	0.601	0.361	0.433	0.069		0.681	0.464	0.587	0.126
ADJ_DRP	0.609	0.371	0.340	0.031		0.682	0.465	0.473	0.008

Accuracy (* r*); theoretical relibability ($\:r^{2}$); estimated reliabilities mean ($\:\overline{{REL}}$); multitrait model (MT); system using deregressed proofs (DRP); system using adjusted deregressed proofs (ADJ_DRP)

### Behavior of EBV from MT, DRP and ADJ_DRP across datasets

**Fig. 3:**
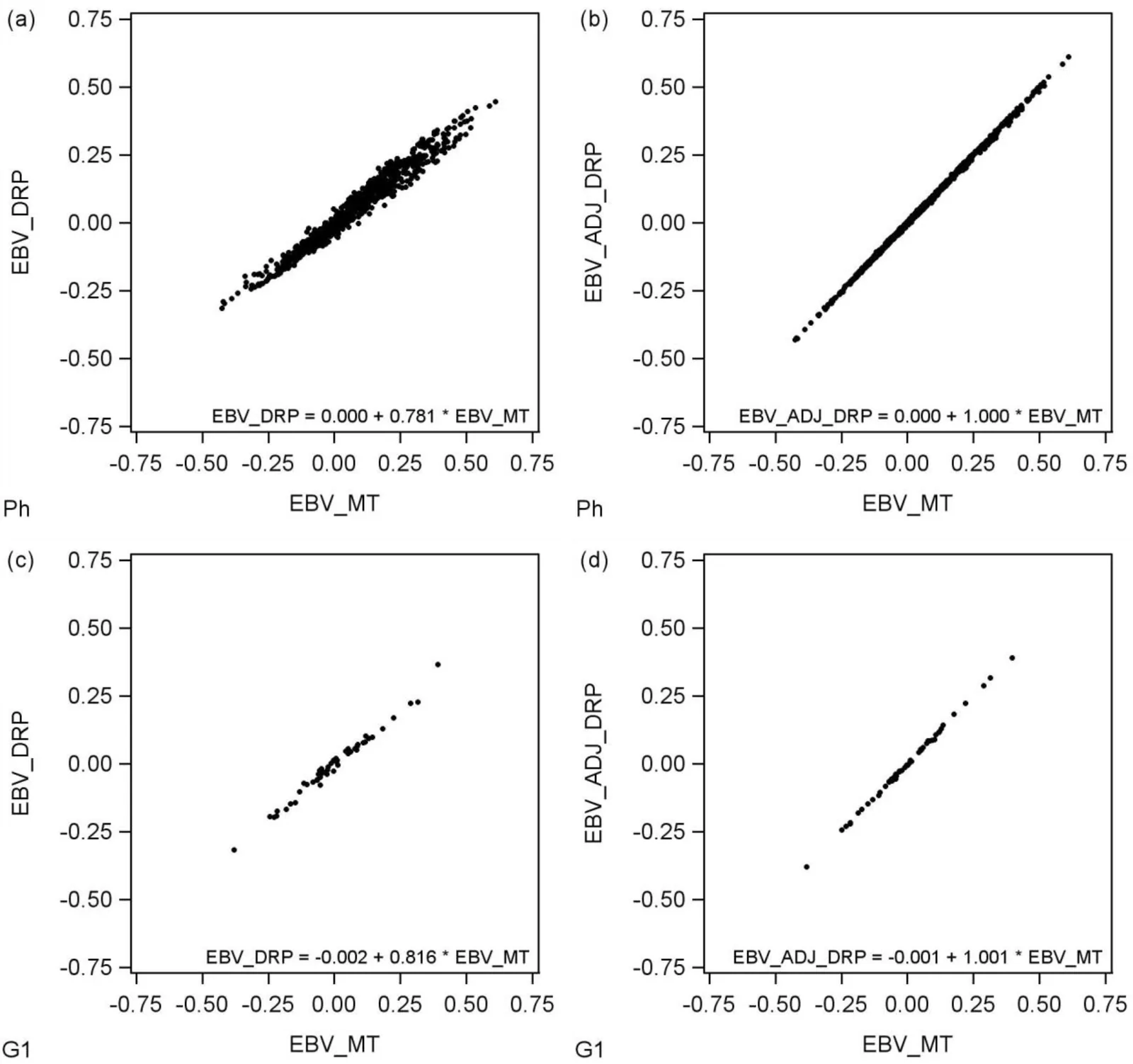
Regression plots of estimated breeding values of real dataset through the three systems. Relationships between estimated breeding values for the target trait from the multitrait model and (**a**, **c**) deregressed proofs (**b**, **d**) adjusted deregressed proofs for phenotyped animals and sires of generation 1 of the real dataset. Abbreviations: estimated breeding values of the target trait in multitrait model (EBV_MT); estimated breeding values of deregressed proofs (EBV_DRP); estimated breeding values of adjusted deregressed proofs (EBV_ADJ_DRP); phenotyped animals (Ph); sires of generation 1 (G1)

**Fig. 4:**
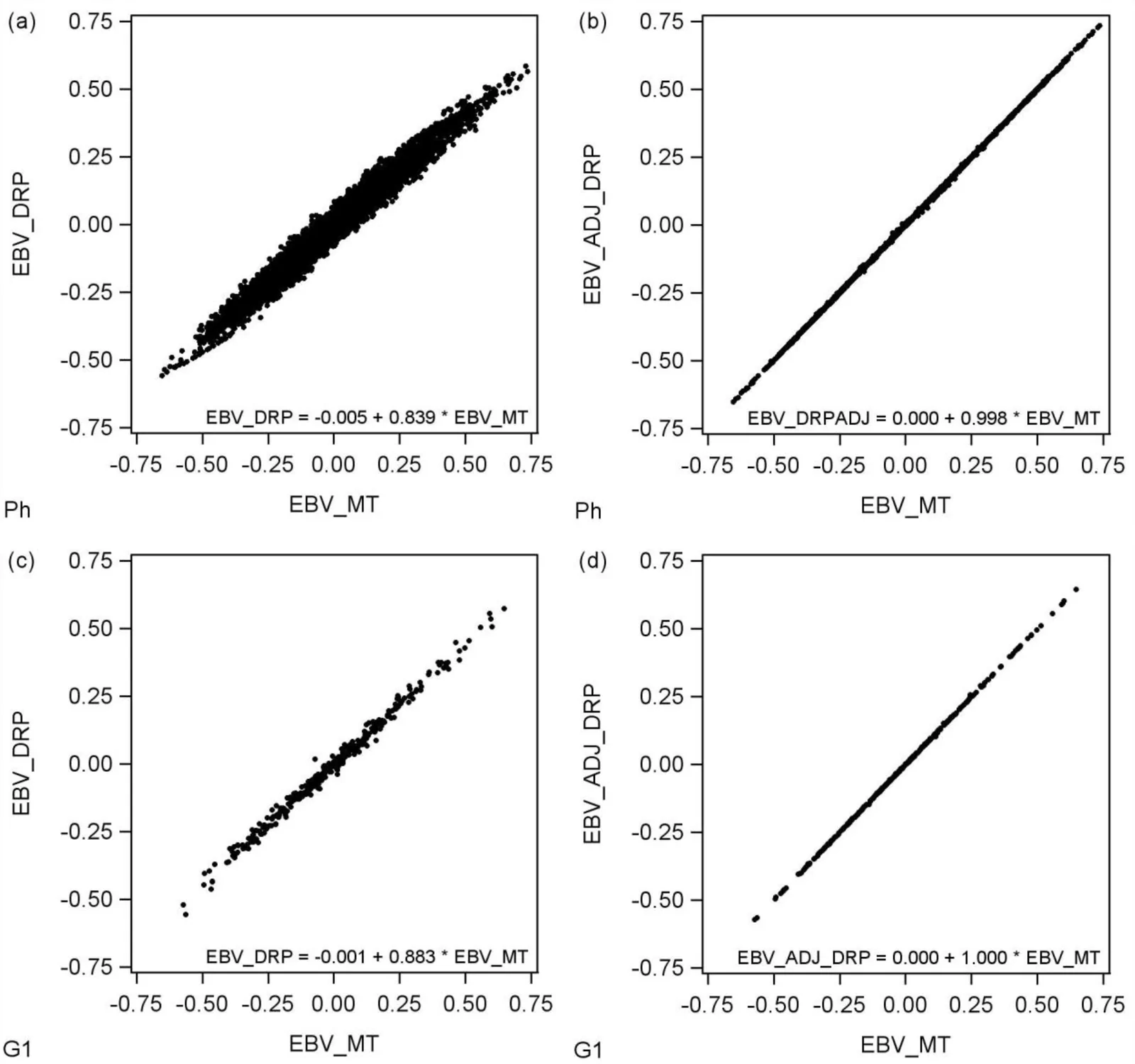
Regression plots of estimated breeding values in the Case 1 through the three systems. Relationships between estimated breeding values for the target trait from the multitrait model and (**a**, **c**) deregressed proofs (**b**, **d**) adjusted deregressed proofs for phenotyped animals and sires of generation 1 of the Case 1. Abbreviations: estimated breeding values of the target trait in multitrait model (EBV_MT); estimated breeding values of deregressed proofs (EBV_DRP); estimated breeding values of adjusted deregressed proofs (EBV_ADJ_DRP); phenotyped animals (Ph); sires of generation 1 (G1)

**Fig. 5:**
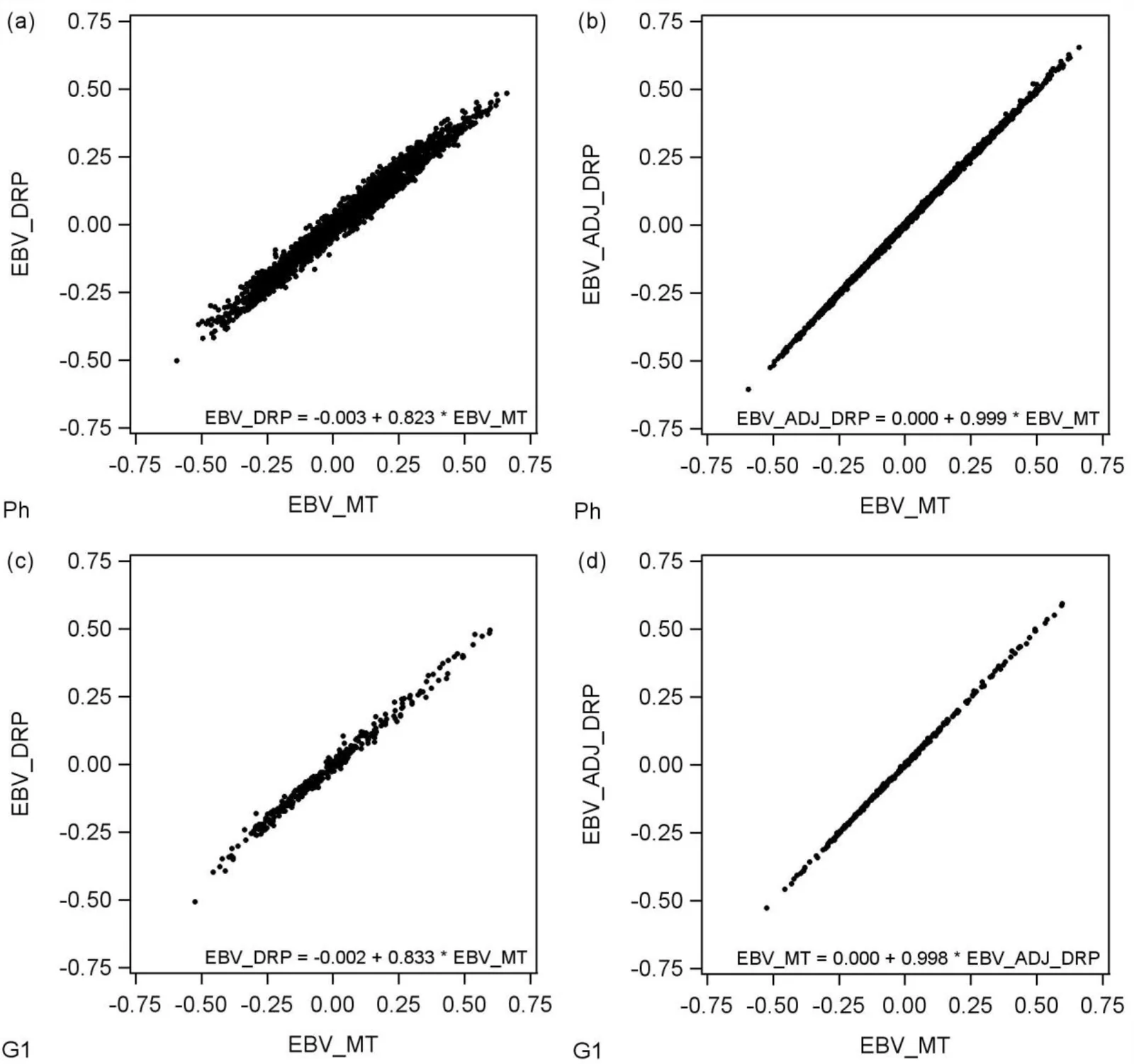
Regression plots of estimated breeding values in the Case 2 through the three systems. Relationships between estimated breeding values for the target trait from the multitrait model and (**a**, **c**) deregressed proofs (**b**, **d**) adjusted deregressed proofs for phenotyped animals and sires of generation 1 of the Case 2. Abbreviations: estimated breeding values of the target trait in multitrait model (EBV_MT); estimated breeding values of deregressed proofs (EBV_DRP); estimated breeding values of adjusted deregressed proofs (EBV_ADJ_DRP); phenotyped animals (Ph); sires of generation 1 (G1)

**Fig. 6:**
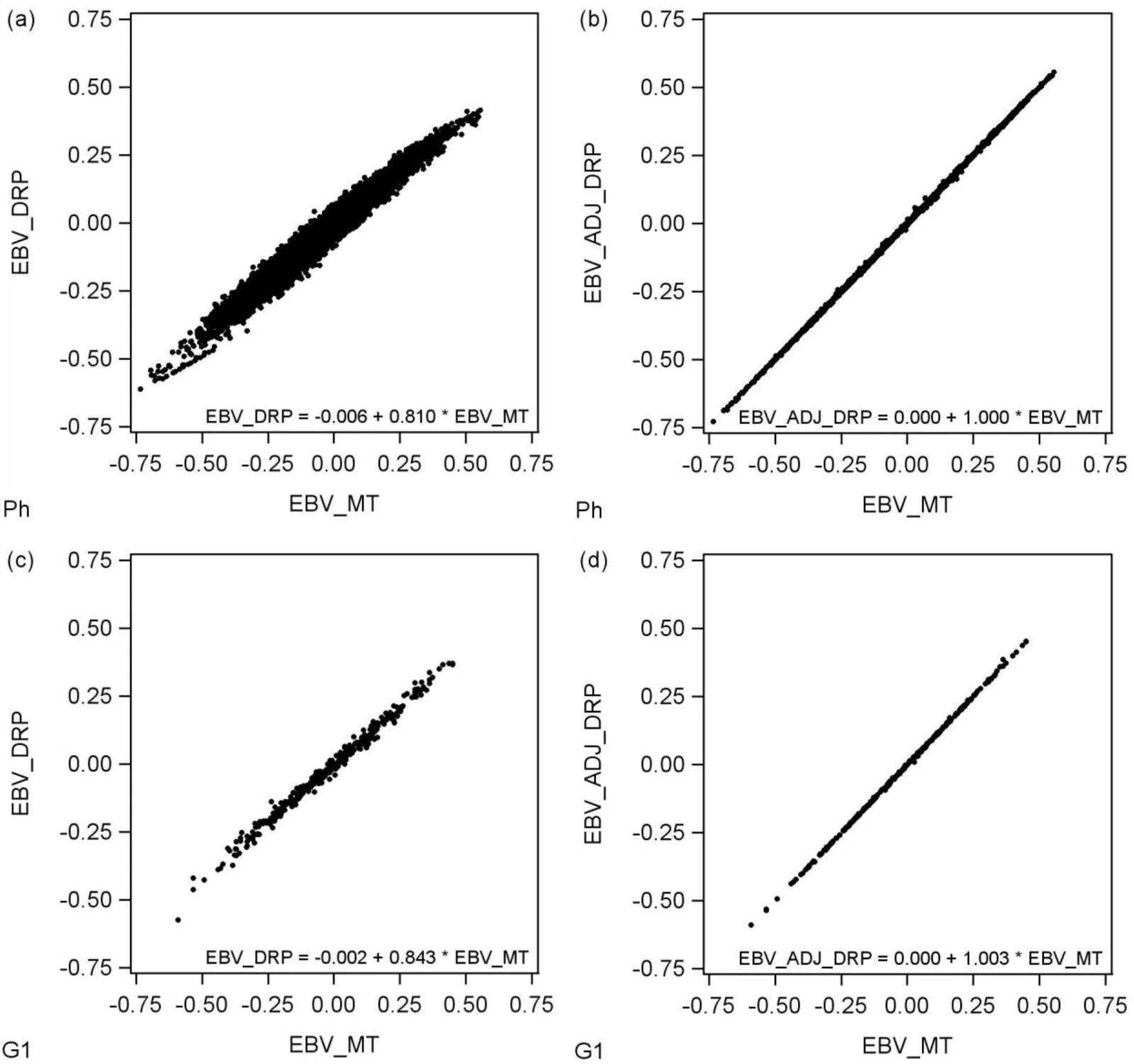
Regression plots of estimated breeding values in the Case 3 through the three systems. Relationships between estimated breeding values for the target trait from the multitrait model and (**a**, **c**) deregressed proofs (**b**, **d**) adjusted deregressed proofs for phenotyped animals and sires of generation 1 of the Case 3. Abbreviations: estimated breeding values of the target trait in multitrait model (EBV_MT); estimated breeding values of deregressed proofs (EBV_DRP); estimated breeding values of adjusted deregressed proofs (EBV_ADJ_DRP); phenotyped animals (Ph); sires of generation 1 (G1)

Figures 3, 4, 5 and 6 show regression plots and illustrate the behavior of EBV_DRP and EBV_ADJ_DRP compared to the reference EBV_MT for phenotyped animals (Ph) and for the sires of G1. Across all datasets and scenarios, EBV_ADJ_DRP regression slopes on EBV_MT were near 1 and intercepts near 0, indicating that EBV_ADJ_DRP effectively reproduced EBV_MT. In contrast, DRP regression slopes on EBV_MT were compressed (0.781–0.883), indicating a deviation of the EBV_DRP from EBV_MT.

### Distribution of EBV across MT, DRP and ADJ_DRP and datasets

Table 4 summarizes EBV obtained from the three evaluations (MT, DRP, ADJ_DRP) for phenotyped animals and G1 sires across the real dataset and the three simulated cases. Clear differences appeared in EBV variability. For phenotyped animals, EBV_DRP were more shrunken, with SD systematically lower than those from MT in all datasets (e.g., 0.119 vs. 0.150 in the real dataset; 0.142–0.169 vs. 0.171–0.199 in the simulated cases). In contrast, ADJ_DRP restored EBV dispersion to levels nearly identical to MT across all scenarios (e.g., 0.149 in the real dataset; 0.170–0.198 matching the MT range in all simulated cases).

**Table 4:** Summary of EBV of the target trait across the three systems for phenotyped animals and for sires of animals with phenotypes for the real dataset and simulated cases

Dataset and Method	Phenotyped animals			Sires	
	EBV Mean	EBV SD		EBV Mean	EBV SD
Real					
MT	0.018	0.150		-0.001	0.143
DRP	0.014	0.119		-0.003	0.118
ADJ_DRP	0.019	0.149		-0.002	0.143
Case 1					
MT	-0.017	0.199		-0.014	0.231
DRP	-0.019	0.169		-0.014	0.205
ADJ_DRP	-0.017	0.198		-0.014	0.231
Case 2					
MT	-0.025	0.171		-0.017	0.209
DRP	-0.024	0.142		-0.016	0.175
ADJ_DRP	-0.025	0.170		-0.017	0.209
Case 3					
MT	-0.021	0.184		-0.011	0.204
DRP	-0.023	0.152		-0.011	0.173
ADJ_DRP	-0.021	0.184		-0.011	0.205

Distribution of phenotyped animals and their sires (1^st^ generation, G1) in the real dataset (*n* = 2169 and *n* = 53) and simulated cases 1–3 (*n* = 9590 and *n* = 307, respectively)

Abbreviations: estimated breeding values (EBV); multitrait model (MT); system using deregressed proofs (DRP); system using adjusted deregressed proofs (ADJ_DRP)

Similar results appeared for sires: EBV_DRP displayed narrower distributions than EBV_MT (e.g., 0.118 vs. 0.143 in the real dataset; 0.173–0.205 vs. 0.204–0.231 in the simulated cases) whereas EBV_ADJ_DRP reproduced MT-levels SD across all scenarios (e.g., 0.143 in the real dataset; 0.205–0.231 in simulated cases).

### Accuracy and reliability coherence across MT, DRP and ADJ_DRP and datasets

In all simulated scenarios, accuracy metrics confirmed the consistent behavior of the three evaluations systems (Table 3). For both phenotyped animals and first-generation sires, using results of Case 3 of phenotyped animals as example here, the MT and ADJ_DRP systems produced equivalent correlations (* r*) and, therefore, theoretical reliabilities ($\:r^{2}$) (e.g., $\:r\:=\:0.609$ and $\:r^{2} \:=\:0.371$), while the DRP system gave slightly lower correlations between true and predicted breeding values (e.g., $\:r\:=\:0.601$). The differences between the reported average reliabilities and theoretical expectations ($\:|\overline{{REL}}\mathrm{-}r^{2} |$) were small for MT and ADJ_DRP (e.g., 0.001 and 0.031) and systematically larger for DRP (e.g., 0.069), indicating biased estimation of the information content by unadjusted deregression.

## Discussion

This study shows that the pooling process based on adjusted DRP accurately reproduces the EBV obtained with an MT model, both in terms of genetic scale and REL accuracy.

A key step in our demonstration was to establish variance components of a real dataset and then extend this to a simulated dataset that replicates the real population variability to study different potential scenarios that may occur in scientific research. The estimated genetic parameters from the real dataset are consistent with earlier studies on boar taint traits. The heritabilities of skatole and androstenone are moderate to high, in agreement with previous findings [15, 17]. The sensory score exhibits slightly lower heritability than found in other studies [7, 17] but still provide relevant additive genetic information for the evaluation. The simulation procedure was successfully performed to extend the real dataset reproducing the variance components considering Frobenius norm results.

The observed convergence of adjustment factors shows the stability and robustness of the iterative adjustment. Although required slightly more iterations in the Case 3, which can be explained by the absence of the target trait, the iterative process achieved satisfactory convergence. Both adjustments are able to reach equilibrium even under the most heterogeneous data configurations.

Across all datasets, comparisons between the MT evaluation, the original DRP and the ADJ_DRP demonstrate clear differences as shown by Figs. 3, 4, 5 and 6; Table 3. Although EBV means remain similar across systems within each scenario, the DRP based values consistently display reduced variability. This confirms the excessive shrinkage introduced by the unadjusted deregression. In contrast, adjustment restores the expected EBV dispersion and produces SD values closely aligned with those of the MT evaluation for the target trait. Thus, EBV_ADJ_DRP accurately reproduce the scale, variability and expected genetic values, fully reflecting the EBV obtained in MT highlighting the importance of the expansion of DRP. This restoration of the scale was obtained by the iterative adjustment of the expansion factor, correcting the level of shrinkage applied to the DRP. Simultaneously with the adjustment of dERC, we ensure that the average information content was properly aligned. The excessive shrinkage observed in our “classical” EBV_DRP as we implemented it, may also be more common in other deregression contexts than previously recognized. Indeed, all DRP independently from the used approach are predictions therefore subject to this behavior, as reported by VanRaden et al. [29]. This issue is not specific to the present application. Indeed, the generated pseudo-phenotypes remain always expected predicted values and may not guarantee that the generated distribution reflects the real values. Even implementations such as those by Jairath et al. [24] only regenerate expected EBV under the hypothesis that the deregression model is also the evaluation model generating the EBV. This latter point is an extension of the issue we solved in our study (i.e., MT model but deregressed EBV are evaluated in ST).

Accuracy results in simulated cases support these observations as reported in Table 4. Correlations between true breeding values and predicted EBV are systematically highest and similar for EBV_ADJ_DRP and EBV_MT, while EBV_DRP have reduced correlations. When focusing on sires of the first generation, a slight trend toward REL overestimation persists in the last simulated case, even after adjustment. However, patterns between MT and ADJ_DRP remain closely aligned and followed similar behaviors. The ranking of sires, in terms of information content, was largely preserved, implying that such deviation would have minimal impact on routine evaluations. REL must be interpreted in terms of their effect on ranking, not their absolute numerical value [39].

Together, these results provide a coherent picture of how the proposed approach integrates MT information through a deregression‑based pooling strategy. The proposed deregression‑based approach effectively concentrates MT information into a single genetically meaningful pooled phenotype and offers greater operational flexibility and is preserving both accuracy and reliability coherence which could be called “extended reversibility” in our context. Indeed, the use of $\:\mathrm{DR}{\mathrm{P}}^{*}$ with $\:\mathrm{dER}{\mathrm{C}}^{*}$ yields the same EBV as the use of the original data (i.e., recorded or simulated in this study) [26]. This extended reversibility is a desired property of the deregression here, as coherence between the REL of EBV obtained from the adjusted deregression and those from the MT evaluation is also required. The target trait could be recorded sparsely or even missing, demonstrating how correlated traits contribute to enhance the genetic information in heterogeneous recording systems.

The proposed framework implies that the pooled phenotype can only recover and transfer the information already present in the MT evaluation used to generate the EBV of the target trait, which constitutes an inherent constraint of the proposed approach. Consequently, the usefulness of the approach is directly dependent on the prediction accuracy of the target trait in the underlying MT model. Case 3 illustrates a particularly extreme situation, characterized by the complete absence of direct measurements of the target trait. Despite this constraint, the moderate-to-high genetic correlations observed with the proxy traits (cf. Table 2) enabled the MT model to provide meaningful EBV. However, for traits characterized by low heritability and weak genetic correlations with available proxy traits enhancing the prediction of the target trait, the MT prediction accuracy of the missing target trait would be expected to decrease substantially. The adjustment factors $\:1/\omega\:$ and $\:\gamma\:$ restore scale and consistency between EBV and REL, but they are limited to the genetic information already known from the initial MT prediction.

### Perspectives

The proposed pooling approach offers several possibilities for future improvements and applications. Beyond individual‑level implementations, this method could be adapted to parent‑level implementations. It would work in a similar way deregressing the EBV based on daughters, transforming it into ERC associated to sire-DRP [22]. For example, this method is currently used when national evaluations recover Multiple Across Country Evaluation (MACE) results from INTERBULL in their national evaluation [40]. A sire‑DRP version of the adjusted deregression process could aggregate MT progeny information which is currently ignored. In general, the extension of the adjusted deregression process to other situations could be considered as long as any double-counting (e.g., no simultaneous deregression of progeny and sire effects) can be avoided.

Although several data configurations were investigated, more complex structures, such as increased numbers of traits, alternative model specifications, additional effects (e.g. permanent or maternal) or complex phenotyped subpopulations, may introduce additional challenges. For example, the implications of our method extend well beyond the context of a few traits analyzed in this study. It can be extended to emerging high‑dimensional phenotyping systems based on sensor technologies, multi‑omic measurements or other heterogeneous data sources, supporting the development of new classes of pooled phenotypes. For example, since the needed information are EBV and REL for the target trait, novel and advanced alternative MT methods as those presented by Runcie et al. [10], which are well suited for high‑dimensional data, could be used.

More broadly, the framework shows strong potential for generalization to situations for which the target trait is sparse or inconsistently recorded. Moreover, the simulated results indicate that it remains applicable even when the target trait is poorly measured or missing, provided correlated traits are available as for the Case 3. Future studies should investigate the operational limits of the method under a broader range of genetic parameters. In particular, simulation studies varying heritability levels, genetic correlations and recording intensities could be used to characterize the minimum MT prediction accuracy required for the pooled phenotype to remain useful for selection purposes. Such studies and practical uses would allow the establishment of knowledge in which situations the pooling process is expected to provide substantial benefits and in which situations the available information is insufficient to generate relevant pseudo-phenotypes.

The adjusted deregression process developed in this study was not yet formulated within a genomic evaluation framework. This reflects the fact that the underlying scalar deregression theory has only recently been revisited and extended to genomic predictions [26]. Future research should therefore focus on integrating genomic information into the proposed framework.

Finally, pooled phenotypes derived from the adjusted deregression process could be integrated into an evaluation system where the target trait is also already available for other records. In this case, their integration with direct observations requires appropriate fixed-effect modelling to account for differences between data sources. In particular, different classes of phenotypes are expected to differ in mean level and must therefore be distinguished using source-specific fixed effects. This adaptability to different data structures supports its applicability in diverse recording and evaluation environments. For example, historically available phenotypic records, collected over multiple years, may be jointly evaluated in routine genetic and genomic evaluation frameworks, including single-step Genomic Best Linear Unbiased Prediction (ssGBLUP), with pseudo-records generated through the use of the adjusted deregression process on data from projects and other advanced phenotyping initiatives.

## Conclusions

We introduced a novel deregression‑based approach to pool heterogeneous MT information into a single pseudo‑phenotype for a given target trait suitable for routine evaluations and validated it in a crossbred-progeny testing scheme. Our results demonstrated that DRP, although preserving a part of the MT signal, produce overshrunk EBV and inconsistent reliability patterns, limiting their suitability. In contrast, the adjusted deregression process successfully restored extended reversibility with the underlying MT model, particularly with respect to EBV scale and distribution, confirming that the adjustments effectively correct the shrinkage issue. Simulation scenarios showed that the method remains effective even when the main trait is sparsely recorded or missing, as long as the correlated traits provide sufficient predictive information for the target trait through the MT evaluation. Although illustrated here on boar taint traits, the approach remains robust across diverse recording patterns, supporting its applicability to other complex phenotypes with heterogeneous or limited phenotyping. Overall, the adjusted deregression process could offer a practical solution for introducing complex, partially recorded phenotypes into standard genetic and genomic evaluation pipelines.

## Data Availability

The datasets are available by contacting authors on reasonable request and with permission of the breeding organizations.
